# Editorial: Myeloid Derived Suppressor Cells as Disease Modulators

**DOI:** 10.3389/fimmu.2020.00090

**Published:** 2020-01-31

**Authors:** Olivera J. Finn, Augusto C. Ochoa

**Affiliations:** ^1^School of Medicine, University of Pittsburgh, Pittsburgh, PA, United States; ^2^School of Medicine, Louisiana State University, New Orleans, LA, United States

**Keywords:** myeloid-derived suppressor cells (MDSC), cancer, immunosuppression, arginase 1 (Arg-1), chronic inflammatory diseases

Myeloid cells are a diverse family of innate immune cells with enormous functional plasticity stemming in part from the lack of genetically encoded antigen-specific receptors. Monocytes, dendritic cells and the various forms of polymorphonuclear granulocytes (eosinophils, basophils, and neutrophils) play fundamental roles in our defense against infectious agents. However, in chronic inflammatory conditions such as cancer, chronic infections, obesity, trauma and chronic stress, myeloid cells become chronically activated, develop mechanisms that suppress T cell, B cell, and even NK cell functions and have thus been named myeloid-derived suppressor cells (MDSC) (1). Similar to their normal counterparts MDSC can be monocytic (M-MDSC) or granulocytic (PMN or G-MDSC) and display a wide array of immunosuppressive mechanisms (2–4). In cancer, where they have been most extensively studied, MDSC can be detected early on in the malignant microenvironment (5) and increase in circulation as the tumors progress. Increases in the numbers of circulating MDSC have been associated with a decreased response to check-point immunotherapies and poor overall survival (6, 7).

The signals and mechanisms that activate and regulate normal myeloid cell function are primarily pathogen-associated molecular patterns (PAMPs) from infectious agents and damage-associated molecular patterns (DAMP's) from damaged tissues. The elimination of the infectious agent or the repair of tissues ends the response of myeloid cells which return to a quiescent stage. In contrast, diseases characterized by chronic inflammation and/or persistent tissue damage such as cancer, autoimmunity, or chronic infections, result in the prolonged release of DAMP's and PAMP's and the production of cytokines such as G-CSF, GM-CSF, and IL6 that increase the release of myeloid-cells from bone marrow and promote the induction of immunosuppressive mechanisms in MDSC. More recently new data show that increased concentrations of lipids such as found in obese patients (8, 9), or increased levels of catecholamines as in chronic pain or stress also promote the activation of immunosuppressive mechanisms by MDSC (10). MDSC suppress T and NK cell function through multiple mechanisms. The depletion of amino-acids such as arginine and L-tryptophan by Arginase I and Indoleamine 2,3-dioxygenase (IDO) induces T cell anergy, while an increased uptake of cysteine by MDSC depletes this amino-acid that is essential for T cell function. The production of reactive oxygen species (ROS) and reactive nitrogen species (nitric oxide—NO) induces T cell apoptosis, while the release of immunosuppressive cytokines such as IL10 and TGFβ, or the production of adenosine inhibit T and NK cell functions. Finally the expression of check-point molecules such as PD-L1 leads to T cell exhaustion, while Fas L and Galectin 9 cause T cell apoptosis. The end result is the loss of protective or therapeutic T cell responses and the escape of tumors from the immune response or the therapeutic effect of novel immunotherapies.

MDSC are therefore the focus of intense research aimed at identifying signals that increase and activate MDSC, understanding their role in different diseases, establishing unique markers that allow us to track the number and fate of these cells, and finding therapeutic approaches to block their immunosuppressive activities. The publications that are part of the series on Myeloid Derived Suppressor Cells as Disease Modulators present original articles and reviews that update on the recently acquired knowledge of the mechanisms involved in the induction and function of MDSC in cancer and other diseases and discuss therapeutic approaches being tested for modulating their function with the goal of allowing the development of a protective T cell functions that resolve the disease process.

## Author's Note

The authors selected and invited the scientific contributors to this collection based on their unique and pioneering discoveries on the role of MDSC in a variety of diseases, the biology of MDSC, their impact on the function of other immune cells and their effect on disease outcomes. We expect that the knowledge presented in these articles provides information for other researchers in the field and eventually helps develop novel therapeutic approaches to regulate the function of MDSC for the benefit of patients.

## Author Contributions

All authors listed have made a substantial, direct and intellectual contribution to the work, and approved it for publication.

### Conflict of Interest

The authors declare that the research was conducted in the absence of any commercial or financial relationships that could be construed as a potential conflict of interest.
